# The costs of late detection of developmental dysplasia of the hip

**DOI:** 10.1007/s11832-014-0599-7

**Published:** 2014-06-29

**Authors:** Timothy Woodacre, A. Dhadwal, T. Ball, C. Edwards, P. J. A. Cox

**Affiliations:** 1 Royal Devon and Exeter Hospital, Barrack Road, Exeter, Devon EX2 5DW UK; 2 17 Halwyn Place, Truro, Cornwall TR1 2LA UK

**Keywords:** Developmental hip dysplasia, Screening, Economics, Ultrasound

## Abstract

**Purpose:**

Debate currently exists regarding the economic viability for screening for developmental dysplasia of the hip in infants.

**Methods:**

A prospective study of infant hip dysplasia over the period of 1998–2008 (36,960 live births) was performed to determine treatment complexity and associated costs of disease detection and hospital treatment, related to the age at presentation and treatment modality. The involved screening programme utilised universal clinical screening of all infants and selective ultrasound screening of at-risk infants.

**Results:**

One hundred and seventy-nine infants (4.8/1,000) presented with hip dysplasia. Thirty-four infants presented late (> 3 months of age) and required closed or open reduction. One hundred and forty-five infants presented at < 3 months of age, 14 of whom failed early Pavlik harness treatment. A detailed cost analysis revealed: 131 early presenters with successful management in a Pavlik harness at a cost of £601/child; 34 late presenters who required surgery (36 hips, 19 closed/17 open reductions, one revision procedure) at a cost of £4,352/child; and 14 early presenters with failed management in a Pavlik harness requiring more protracted surgery (18 hips, four closed/14 open reductions, seven revision procedures) at a cost of £7,052/child.

**Conclusions:**

Late detection causes increased treatment complexity and a sevenfold increase in the short-term costs of treatment, compared to early detection and successful management in a Pavlik harness.

**Discussion:**

Improved strategies are needed for the 10 % of early presenting infants who fail Pavlik harness treatment and require the most complex and costly interventions.

## Background

We have assessed the cost of both our current screening and treatment programmes for developmental dysplasia of the hip (DDH), and have modelled the costs of alternate screening strategies.

DDH is a common and preventable cause of childhood disability [1], with a quoted incidence between 1.4/1,000 and 20/1,000 live births [1–3]. Readily identifiable risk factors exist, including breech position, first birth and a positive family history [1, 4]. DDH can be detected clinically, including by the combined Ortolani–Barlow manoeuvre, which has a quoted sensitivity and specificity of 7–98 and 84–99 %, respectively [5–7]. Ultrasound scanning (USS) is the gold standard for detecting DDH in the neonate, with a sensitivity of 100 %, although it can over-diagnose the condition (labelling natural hip immaturity as dysplasia) [8, 9]. Unrecognised or mistreated DDH can result in long-term hip deformity and morbidity [10]. Early diagnosis whilst the hip and acetabulum are maturing increases the chance of successful nonoperative management in abduction devices, such as the Pavlik harness. Late diagnosis, commonly accepted as > 3 months, increases the likelihood of surgical intervention being required [1, 10]. DDH is therefore an ideal condition to screen for.

### Screening programmes

Screening programmes for DDH have existed since the 1930s. Different programs include the pure clinical examination of neonates, selective USS of at-risk neonates and universal neonatal USS. Due to the lower sensitivity and reliance on clinician skill, screening programs based purely on clinical examination have a lower rate of early identification of DDH, resulting in an increased incidence of late presentation and surgical management. USS screening incurs more time and cost than pure clinical screening; however, its supporters maintain that the additional cost is offset by the reduced incidence of late presenters and interventional treatment [11, 12]. Recent arguments have been made to abandon screening altogether, due to the time and cost incurred by any DDH screening program relative to the number of favourable outcomes achieved. However, an analysis by Sewell and Eastwood [13] highlighted that this would significantly increase the rates of late detection, the rates of avascular necrosis (AVN) secondary to open surgical reduction, and the requirement for femoral and acetabular osteotomy.

### Cost analysis

Limited detailed data exists to provide economic support for or against DDH screening and the different screening programs available. The multicentre UK Hip Trial reviewed 629 patients and suggested a decrease in cost of £100 per patient when USS was used in screening, yet failed to demonstrate statistical significance [14]. Thaler et al. [11] promoted universal USS screening by demonstrating a significant reduction in cost of all surgical and non-surgical treatments for DDH secondary to its introduction in Tyrol Austria, accompanied by an increase in annual screening cost of Euro £57,000. The most comprehensive financial review was performed by Cleg et al. [12]. This assessed 20 years of experience in Coventry UK, where the cost of screening reduced from £5,110 to £3,811 per 1,000 live births following the implementation of universal USS screening. The number of patients requiring surgical intervention, and more extensive surgery, decreased. There is, however, still no clear data detailing the exact costs of different screening programs relative to increased favourable outcomes.

### Current UK guidance

The NHS Newborn and Infant Physical Examination (NIPE) Programme stated in their 2010 guidance that universal USS screening was not recommended, but that there should be selective USS screening based upon risk factor association and universal neonatal clinical examination [15]. A child with an abnormality elicited by neonatal clinical examination should undergo an USS within 4 weeks. A child with a normal neonatal clinical examination yet with risk factors for DDH should undergo an USS at 6 weeks.

### Current regional DDH screening programme

Our region currently provides universal clinical screening, and selective USS screening of children with risk factors for DDH. All neonates undergo a hip examination within 48 h of birth by a trained member of the paediatric team and at 6 weeks by a trained member of the community team (general practitioner or health visitor). All neonates with clinical suspicion of DDH are referred to the regional dysplasia clinic. All neonates with a breech delivery or breech presentation subsequent to 36 weeks gestation or with a positive family history of DDH are referred for USS assessment at 6 weeks age and subsequent referral to the regional dysplasia clinic if DDH is detected.

The regional dysplasia clinic consists of a consultant paediatric orthopaedic surgeon, a sonographer and a senior physiotherapist, all with a specialist interest and relevant training in DDH. All neonates undergo clinical examination and USS scanning with Graf assessment of DDH pathology on static US images and additional dynamic USS. Pavlik harness management is used for infants with confirmed DDH who are subsequently followed up at bi-monthly clinics for USS assessment of hip reduction within harness, with escalation of treatment to surgical intervention if reduction within a Pavlik harness fails.

## Aims

We aimed to perform a comprehensive cost analysis of our current regional model for DDH screening. The primary outcome measure was to determine treatment complexity and associated costs of DDH detection and hospital treatment, related to the age at presentation and treatment modality. The secondary goal was to model the costs of alternate screening strategies relative to the number of additional favourable outcomes.

## Methods

The Regional Live Birth Data and the STORK neonatal database were analysed to gain relevant regional epidemiological information.

### Analysis of current selective screening programme

The regional screening programme in its current form was introduced in 1997. All children with confirmed DDH had relevant information regarding their background, referral modality and treatment prospectively entered onto a secure dysplasia database. We analysed the database from 1997 to 2008. This provided an 11-year review of the screening and treatment of regional infant hip dysplasia.

#### Inclusion criteria

All children with confirmed DDH referred to the dysplasia clinic were analysed.

#### Exclusion criteria

- Children referred from out of region, where part or all of their screening and previous treatment had been completed separate from the regional screening program.
- Children with an associated neuromuscular disorder thought to contribute towards DDH, due to differences in age of presentation, detection and treatment modalities.

### Cost analysis of current screening programme

Costs were based on 2008 prices and were determined for:

- Neonatal clinical examination screening (at birth and 6 weeks post-partum)
- USS screening
- Dysplasia clinic assessment and Pavlik harness management
- Inpatient surgical management
- Outpatient follow-up

Costs included:

- Personnel cost (wages relating to time per patient/clinic)
- Consumable cost
- Administrative support
- Overheads

Cost analysis was divided into

1. The screening of normal children without hip dysplasia
2. Children presenting early (< 3 months age), allowing treatment with a Pavlik harness
3. Children presenting late (> 3 months age) requiring surgical intervention.

### Modelling the costs of alternate screening strategies

The total cost of screening and treatment of DDH over an 11-year period was broken down into its constituent components. Accurately determined costs for each component were then used to model the costs of alternate screening strategies:

- Current selective USS screening of “at-risk” live births
- Universal USS screening of all live births
- USS screening of all female live births plus male “at-risk” live births.

## Results

### Outcome of current selective screening program

Following use of the current screening programme, analysis of all referrals from 1997 to 2008 demonstrated:

- 36,960 live births screened for risk factors and via clinical exam.
- 280 children were referred to the regional dysplasia clinic with confirmed DDH. There were 101 exclusions, generating 179 appropriate for study.
- The 179 children demonstrated 242 dysplastic hips. The nature of hip pathology observed is characterised in Table 1.

**Table 1:** Characterisation of hip pathology for treatment groups

Group	No. pts	No bilateral DDH	No unilateral DDH	Mean age presentation days (+ range)	Graf 2c hips	Graf III hips	Graf IV hips	X-ray abnormal^a^
Pavlik success	131	54	77	17 (1–90)	38	131	16	–
Pavlik fail	14	7	7	26 (1–90)	0	4	17	–
Late presentation	34	2	32	486 (82–1,955)	2	0	6	28
Totals	179	63	116	–	40	135	39	28

^a^28 hips had radiographic evidence of dislocation or marked subluxation
- 81 % (*n* = 145) were successfully identified by the screening programme and presented early (before 3 months of age).
- 19 % (*n* = 34) were not identified and presented late secondary to clinical concern in the community (limited hip abduction, limb length discrepancy or gait abnormality).
- The 145 children presenting early demonstrated 168 dislocated (Graf III or IV) hips and 38 critical range dysplasia (Graf 2c) hips. These were all treated in Pavlik harnesses. This proved successful for 90 % (*n* = 131). Therefore, there was a 90 % success rate for Pavlik harness management of those children where DDH was detected early, before 3 months of age, regardless of Graf classification.
- There was a 10 % failure rate of managing all children presenting early via a Pavlik harness, requiring subsequent surgical intervention. This failure of hip reduction or retention in harness was only observed with Graf IV hips.
- In 34 children who presented late (over 3 months of age) both neonatal and 6-week hip examinations were thought to be normal and no additional risk factors were present. These children had 36 DDH hips, the majority being confirmed radiographically as either dislocated or subluxed (subsequently confirmed on arthrography). In this group, Pavlik harness was only used as the primary treatment in three patients. In two of these patients, dysplasia remained, requiring further intervention.
- Therefore, 33/34 of late presenting patients had attempted closed reduction under general anaesthesia, and half of these children failed closed reduction and required open reduction as a minimum. Table 2 specifies the type of primary surgical intervention required for the 10 % of early-presenting patients failing Pavlik harness management, and for those patients presenting late. Table 3 specifies the type of secondary surgery required in addition to the primary intervention.

**Table 2:** Characterisation of primary surgical treatment required for hips in each group

Group	Mean age start Rx (days)	Mean Duration Spent in Pavlik Harness (days)	Pavlik	Closed reduction	Open reduction	Pelvic osteotomy	Femoral osteotomy
Pavlik success	33	63	131	0	0	0	0
Pavlik fail	36	30	14	4	16	0	0
Late presentation	373	47	3	15	19	4	2

**Table 3:** Characterisation of secondary surgical treatment required for hips in each group

Group	Revision open reduction	Hip arthrogram	Pelvic osteotomy	Femoral osteotomy	Calculated additional cost^a^
Pavlik success	0	0	0	0	0
Pavlik fail	2	3	5	1	£21,733
Late presentation	0	0	1	1	£5,283

^a^Additional cost = surgical treatment cost plus cost additional outpatient follow up and extra investigations

### Cost analysis

Personnel costs per regional DDH clinic and per patient are summarised in Table 4. The average regional DDH clinic incurred a cost of £538, with cost per patient being £122 per new referral and £83 per follow-up. A breakdown of costs for community surveillance, outpatient treatment and inpatient treatment are summarised in Tables 5, 6 and 7, respectively.

**Table 4:** Breakdown of personnel cost per regional DDH clinic and per patient

Personnel	Experience	Session rate (cost per clinic in £)	Cost per patient (£)	
			First clinic	Follow-up clinic
Consultant	10 years	208.53	46.34	34.76
Nurse	Band 5	48.15	16.05	8.02
Ultrasonographer	Band 7	75.47	25.16	12.58
Physiotherapist	Band 6	52.83	8.81	8.81
Administration	Band 3	30.78	2.56	2.56
Secretary	Band 4	32.74	2.73	2.73
Overheads		89.70	20.33	13.99
Total cost		538.20	121.97	83.35

**Table 5:** A breakdown of costs for surveillance of DDH in the community

Surveillance activity	Cost per patient £
Data collection	2.60
Normal neonatal hip exam	7.32
Abnormal neonatal hip exam	14.73
GP/health visitor hip exam	5.47
Hip ultrasound	56.00

**Table 6:** A breakdown of costs for outpatient treatment

Outpatient Activity	Unit cost £
Pavlik harness	35.00
First regional DDH clinic/harness clinic	121.97
Follow-up regional DDH clinic/harness clinic	83.35
Aftercare physiotherapy	9.36
Consultant follow up outpatient clinic	64.97

**Table 7:** A breakdown of costs for inpatient treatment

Inpatient activity	Unit cost £
Pre-assessment visit	54.85
Inpatient stay: 1 day (for closed hip reduction)	294.07
Inpatient stay: 4 days (for open reduction/osteotomy)	1,167.07
Arthrogram	151.83
Change hip spica	278.56
Abduction casting	174.56
Open reduction	747.91
Pelvic osteotomy	853.93
Femoral osteotomy	1,149.28

### Cost analysis of current selective screening program

A breakdown of the cost of the selective regional screening programme per component parts is demonstrated in Table 8. The 11-year screening programme cost approximately £1.14 million, £104,000 per annum. Approximately 25 % (£243,000) of this was accounted for by the neonatal exam in the community, 20 % (£201,000) by general practitioner (GP) and health visitor checks in the community, 25 % (£244,000) by USS screening, 5 % (£71,000) by Pavlik harness management and 25 % (£238,000) by surgical intervention. Of the annual cost of screening, 73 % (£76,000) was spent demonstrating that 99.5 % (*n* = 3,344) had normal hips, and 27 % (£28,000) was spent demonstrating that the remaining 0.5 % (*n* = 16) were dysplastic.

**Table 8:** A breakdown of costs of the selective regional screening programme

	Number	Number/annum	Index cost	Total 11-year cost	Annual cost
Clinical based surveillance					£54,000
Data collection	36,960	3,360.0	£2.60	£96,096	£8,736
Neonatal exam					
Normal	33,191	3,017.4	£7.32	£242,958	£22,087
Abnormal	3,624	329.5	£14.73	£53,382	£4,853
GP HV exam	36,815	3,346.8	£5.47	£201,378	£18,307
Specialist radiological treatment					£22,000
Ultrasound					
Normal	3,290	299.1	£56.00	£184,240	£16,749
Immature	334	30.4	£140.00	£46,760	£4,251
DDH	145	13.2	£99.48	£12,975	£1,180
Specialist orthopaedic treatment					£28,000
DDH clinic					
Pavlik success	131	11.9	£492.64	£64,536	£5,867
Pavlik fail	14	1.3	£490.15	£6,862	£624
Surgical treatment					
Late presenter	34	3.1	£4,341.71	£147,618	£13,420
Failed Pavlik	14	1.3	£6,428.28	£89,996	£8,181

*HV* Health Visitor

95 % (£21,000) of the annual cost incurred by USS screening was spent proving that referred infants had normal hips. Only 5 % (£1,180) of the cost incurred by USS screening was spent on infants with DDH.

Table 9 demonstrates the cost per child of treatment. Interestingly, whilst the cost of successfully treating early presenters via Pavlik harness was £601 and the cost of treating late presenters was approximately seven times higher (due to increased cost of interventional treatment), the cost of failing to successfully manage early presenters in a Pavlik harness and the subsequent treatment required was approximately 12 times higher. This additional cost was due to the increased time and resources spent attempting to manage within a harness, in addition to the subsequent, more interventional treatment. Table 10 summarises the previous findings and demonstrates the cost per treatment modality, per patient, per presentation per year.

**Table 9:** Cost per child of screening and treating children with DDH relative to mode of presentation

Treatment modality	Mode of presentation	Cost £
(Normal child)	–	22
Pavlik harness	Early	601
Surgery	Late	4,351
Failed Pavlik harness then surgery	Early	7,025

**Table 10:** The cost of clinical examination, USS screening and treatment of all children in the region over an 11-year period

	Normal children (*n* = 36,781)	Pavlik success (*n* = 131)	Pavlik fail (*n* = 14)	Late detection (*n* = 34)	All children (*n* = 36,960)	Per annum (*n* = 3,360)
Clinical hip examination, selective USS screening, harness treatment						
Data collection	95,631	341	37	88	96,097	8,736
Neonatal hip examination	293,963	1,722	196	284	296,166	26,924
6-week hip examination	201,192	717	77	186	202,172	18,379
USS	231,000	11,482	1,271	384	244,137	22,194
Initial assessment clinics	0	42,746	3,713	2,524	48,982	4,453
Physiotherapy input	0	2,023	244	727	2,995	272
Pavlik harness cost	0	6,681	585	160	7,426	675
Hip radiographs	0	0	572	1,399	1,971	179
Follow-up clinics	0	–	996	–	996	91
Subtotal cost	821,786	65,712	7,704	5,787	900,989	81,908
Cost per patient	22	502	544	164		
Surgical treatment						
Index primary surgery	0	0	65,632	106,120	171,752	15,614
Secondary surgery	0	0	11,285	2,375	13,660	1,242
Subtotal cost	0	0	76,917	108,495	185,412	16,856
Cost per patient	0	0	5,494	7,750		
Clinical follow-up						
Clinic appointments	0	10,430	5,050	12,450	27,930	2,539
CT/MRI	0	0	2,384	5,829	8,213	747
Hip radiographs	0	2,576	6,292	15,389	24,258	2,205
Subtotal cost	0	13,006	13,726	33,669	60,401	5,491
Cost per patient	0	99	989	990		
Overall cost	821,786	78,718	98,347	147,950	1,146,801	104,255
Unit cost per patient	22	691	7,025	4,351		

Index primary surgery: hip arthrogram, closed or open reduction, supplemental femoral and pelvic osteotomies, and plaster (spica) changes. Includes the costs of inpatient stay, theatre costs, all personnel costs, consumables, overheads, administration and secretarial costs

Secondary surgery: additional surgery to deal with complications and residual sequelae, e.g., re-dislocation, residual dysplasia

### The costs of alternate screening strategies

Table 11 highlights the key differences in cost between the current screening programme, one that would involve USS screening all girls and at-risk boys, or universal USS screening. The cost of the community exams (which includes the neonatal hip exam) understandably would not change between the programmes as the same number of examinations would be performed. The cost incurred by USS would increase relative to the additional number of neonates scanned in each program (the additional number of girls or the total number of neonates, respectively). The cost incurred by specialist orthopaedic intervention (clinics and surgery) would increase relative to the number of neonates identified early, but also failing early management.

**Table 11:** Breakdown of annual costs of different screening programs

Screening program	Current program	USS of all girls and at-risk boys	Universal USS of all births
Community exam	£54,000	£54,000	£54,000
Specialist radiology	£22,000	£90,400	£207,400
Specialist orthopaedics	£28,000	£18,400	£19,500
Total	£104,000	£162,900	£280,900

Table 12 highlights the number of additional favourable outcomes per screening program (the number of neonates no longer “missed” by the screening program and presenting early rather than late), the additional cost of each program, and thus the additional cost per favourable outcome.

**Table 12:** Additional favourable outcomes and respective costs of different screening programmes

Screening program	Current program	USS of all girls and at-risk boys	Universal USS of all births
Additional annual cost	£0	£58,900	£176,900
Additional favourable outcome per year	0	2.3	3.7
Cost per additional favourable outcome	0	£25,600	£47,800
Late presenters still occurring per year	3	0.4	0

## Discussion

We have demonstrated the successful application of a universal clinical and selective USS screening programme for DDH over an 11-year period. This required inter-trust coordination over a large geographical area and a skilled dysplasia clinic team. Successful Pavlik harness management of patients presenting early (< 3 months) was 90 %, irrespective of initial Graf classification of dysplasia. This is higher than the majority of the published literature [16–18], with only Uçar et al. [19] publishing equivocal success rates for both Graf III and IV hips. A relatively large proportion of Graf IV hips (33/206) was observed in our study compared to that expected from other DDH series [16–18]. All 10 % of children presenting early but failing Pavlik harness management had Graf IV hips. In these children, harness treatment was discontinued at an early stage (to avoid iatrogenic induced Pavlik harness disease).

All infants presenting late required surgical intervention, which concurs with current literature [4].

Comprehensive cost analysis revealed an annual cost of £104,000 to screen 3,360 neonates, identifying and treating early 13 patients, “missing” and treating late three patients. Late detection of DDH caused increased treatment complexity and a sevenfold increase in the short-term costs of treatment, compared to early detection and successful management in a Pavlik harness. However improved strategies are needed for the 10 % of early presenting infants who fail Pavlik harness treatment and require the most complex and costly interventions (twelvefold increase in cost).

The screening programme analysed adheres to UK Newborn and Infant Physical Examination Programme (NIPE) guidelines, providing universal clinical screening and USS screening of patients with abnormal clinical findings or defined risk factors (including positive family history, breech position or structural foot abnormalities). Female neonates are at sevenfold risk of DDH compared to males. Increasing USS screening to include all females and at-risk males would generate a further 2.3 favourable outcomes a year, at a cost of £25,600 per favourable outcome, but would still result in one male infant presenting late every 2 years. Increasing to universal USS screening would double the cost per favourable outcome to £47,800, but would theoretically eliminate infants presenting late. Universal USS screening of 3,360 neonates/year would cost £12 per patient. This is double the cost ascribed by Clegg et al. [12]; however, our cost analysis is more comprehensive than the majority described in the literature, accounting for key additional costs, specifically administrative and overhead costs, and importantly the cost of community GP and health visitor checks.

The study is limited by only analysing the short-term costs of DDH screening in the initial management and follow-up (to discharge) of patients. It does not assess the long-term cost of subtle missed DDH to those patients identified with increasing frequency in young adult hip clinics with pathology secondary to mild acetabular dysplasia, or how increased USS screening would impact on this [20].

The study adds to current literature by providing a detailed analysis of the costs and outcomes of an effective DDH screening programme that adheres to current UK NIPE guidelines. It provides the means to assess the impact of an increased use of USS in screening and to determine the clinical and economic viability of such screening programmes.
